# Investigation of individual strategies in the aerial phase in ski jumping

**DOI:** 10.1038/s41598-023-49683-0

**Published:** 2023-12-15

**Authors:** Petter Andre Husevåg Jølstad, Matthias Gilgien, Ola Elfmark

**Affiliations:** 1 Department of Physical Performance, Norwegian School of Sport Sciences, Oslo, 0863 Norway; 2 Engadin Health and Innovation Foundation, Center of Alpine Sports Biomechanics, Samedan, 7503 Switzerland; 3 Department of Neuromedicine and Movement Science, Centre for Elite Sports Research, Norwegian University of Science and Technology, Trondheim, 7491 Norway; 4 Norwegian Olympic and Paralympic Committee and Confederation of Sports, Oslo, 5000 Norway

**Keywords:** Applied physics, Fluid dynamics

## Abstract

The purpose of this investigation was to examine the performance strategy of three ski jumpers during the steady glide phase and explain how different strategical solutions can lead to jumps of roughly the same length. In this study, a total of 24 jumps performed by two World Cup (WC) athletes and one Continental Cup (COC) athlete were measured with a differential Global Navigation Satellite System (dGNSS) on a large ski jumping hill. For each athlete, the continuous position data, velocity, aerodynamic forces and lift-to-drag ratio (*LD*-ratio) were averaged and compared for the steady glide phase to examine individual jump strategies. The dGNSS yielded accurate measurements of trajectory, velocity and aerodynamic forces, revealing clear differences between the athletes. The largest differences were found between the WC athletes and the COC athlete. The WC athletes focused on maximizing horizontal velocity while the COC athlete minimized vertical velocity. This difference may be explained by the different hill sizes the athletes normally compete on. One of the WC athletes consistently increased their horizontal velocity during the end of the steady glide phase by maintaining a high *LD*-ratio, which highlights the effect of aerodynamics on the resulting velocity, trajectory and jump length.

## Introduction

Ski jumping is a popular winter sport with a rich history and was one of the original disciplines at the first Winter Olympic Games in Chamonix, France^1^. The first known biomechanics research in ski jumping was conducted by Strauman in 1926, and the first known analytical model of mechanics in ski jumping was developed by Strauman in 1927^2^. Since then, the sport has undergone substantial changes with regard to hill size, technique, equipment and, in recent years, technology. Technology is being used on most hills on a daily basis for athlete safety and in competition to measure performance and enhance the viewer experience. This includes take-off speed (measured in the in-run with photocells), wind measurements (measured by anemometers along the hill) and analysis, and laser light marking in the landing area to show the length needed to take the lead in the competition. Technology is also used by teams, coaches and athletes to analyze performance to get a competitive advantage. Performance analysis is no longer done solely by researchers since a range of analytical tools have become more available at consumer level. It has previously been proposed that a considerable amount of knowledge and understanding of biomechanics in ski jumping might not be published due to the competitive nature of the sport^2^, and this is likely to apply now more than ever, since several measurement methods are easily accessible. However, ski jumping has been a popular research topic over the last twenty years^3^, and the primary focus has been on the take-off.

Ski jumping is typically split into four phases: in-run, take-off, aerial, and landing, where the aerial phase is divided into three sub-phases: gliding preparation, steady glide, and landing preparation^4^. The ample space that athletes move through from the in-run to the landing makes it difficult to measure performance variables from all phases, which may be the main reason why most research has focused on the take-off. Take-off is also considered the most critical phase since it dictates the initial conditions of the aerial phase, and because it is a highly complex movement at high speed that is executed over a short period of time^2,5,6^. Several methods have been used to measure performance in ski jumping in the field: force plates have been mounted under the end of the in-run to measure timing and ground reaction force^7–9^, plantar pressure has been measured during take-off and landing using pressure insoles^10–12^, inertial measurement units have been used to measure kinematics and kinetics during different phases^13–16^ and video analysis has captured the motion of the athlete during the early aerial phase^6,17–22^.

Video-based measurement approaches have been the most popular method in ski jumping research, as data can be extracted from competitions without interfering with the athletes. Since the take-off phase is the most critical part of a ski jump and has a limited measurement volume, video analysis is suitable to capture athlete kinematics for that phase^2^. However, the restricted measurement volume captured by camera(s) at take-off does not allow tracking of how differences at take-off propagate through the aerial phase. Video analysis is also usually restricted to linear and angular kinematics, describing the athlete’s technique, trajectory, and velocity, while the aerodynamic forces during the aerial phase are little known. An interesting finding from earlier video-based ski jumping research has been that similar performance can occur with large individual differences in athletes’ technical solutions. This was highlighted by Virmavirta et al.^6^ during a take-off analysis of the normal hill Olympic competition in 2006, where they concluded that the best athletes executed the take-off very differently, yet achieved similar results in the competition. Schmöltzer and Müller^20^ presented similar findings 4 years earlier, where individual glide styles of the ten best athletes in the world were analyzed in the large hill tournament. It was found that the athletes were good at reproducing their glide strategies from jump to jump. In contrast, the difference in strategies between athletes was large, emphasizing that each athlete has their own technical fingerprint in their performance strategy.

Understanding how different individual strategies affect performance is essential to understanding the sport, for both practitioners and researchers. Athletes and coaches strive to gain valid and objective information about athletes’ performance in all parts of a jump since it could give a competitive advantage. As discussed above, video analysis is not very suitable for extracting kinematics and kinetics during the aerial phase^2,18^. A method that allows tracking of athletes from the start of the inrun to the landing is Global Navigation Satellite Systems (GNSS). GNSS was proposed several years ago as a method that could be applied to capture the aerial phase of ski jumping^1^. Since then, GNSS has gained significance in individual sports such as orienteering^23^, sailing^24^, alpine skiing^25–27^ and cross-country skiing^28^, where position and time information and its derivatives are used for instantaneous performance analysis, technique, and tactical analysis. Measurement with sufficiently accurate, industrial grade differential GNSS (dGNSS), was first introduced in ski jumping by Blumenbach^29^. The dGNSS that was used in this study was validated for antenna position (0.05 m)^30^ in alpine skiing and compared to an optical system in ski jumping^3^, revealing that position, velocity and aerodynamic forces were valid during the glide phase but not during take-off. Subsequently, the system was applied in a methodological study to define the start and end of the steady glide phase^4^, and in a comparison of performance variables between a normal and a large hill^31^.

Given the limited research on individual strategies in ski jumping, the aim of this study was to examine the performance strategy of three athletes during the steady glide phase and explain how different jumping strategies can lead to roughly similar jump lengths. The dGNSS measurement system was applied to investigate the steady glide phase on a large hill for jumps performed from the same initial condition (start gate) by three elite athletes. Based on previous research, we hypothesized that the performance variables would be significantly different between athletes, with the most significant dissimilarities being between the athletes at different performance levels.

## Methods

### Protocol

The data collection was carried out in Lysgaardsbakken (HS140, K-point: 123 m) in Lillehammer, Norway. HS140 is a large ski jumping hill regularly used in World Cup (WC) competitions. The data collection was carried out over 3 separate training sessions over a period of 2 days. The coaches characterized all training sessions as having “still wind conditions”. The investigation was based on measurements of three different ski jumping athletes. Two of the athletes had, for several years, participated in World Cup competitions. The third athlete competed in the Continental Cup (COC), the second-highest performance level in ski jumping. The athletes are denoted WC1 and WC2 for WC athletes 1 and 2 and COC1 for the COC athlete. Since the aim of the study was to investigate individual differences for the same initial conditions, all jumps were started from the same gate (9). This was done to exclude factors that could influence the performance when athletes are starting from different gates. Gate 9 was the most commonly used by the athletes. In total, 24 jumps were measured for the three athletes (WC1: 9, WC2: 9, COC1: 6). The study was conducted in accordance with the Declaration of Helsinki^32^, and was approved by the Norwegian Centre for Research Data and the ethical committee of the Norwegian School of Sport Sciences. All athletes signed an informed consent form for participation in the study. The next section provides an overview of the dGNSS method and parameter calculations. For more description of the methodology, the reader is referred to Elfmark et al.^3,4,31^.

### dGNSS measurement

The inrun and the position of the jump’s take-off edge were measured using a dGNSS rover and a base station that both consisted of an antenna (GrAnt-G3T, Javad, San Jose, CA, USA) mounted on a tripod and a receiver (Alpha-G3T, Javad, San Jose, CA, USA) that were positioned at the top of the inrun. The trajectory of the athletes was measured with a dGNSS, with the antenna (G5Ant-2AT1, Antcom, Torrance, CA, USA) mounted on the helmet of the athlete, the receiver (Alpha-G3T, Javad, San Jose, CA, USA) carried in a backpack, and the same base station as for the inrun measurements. The antenna on the athlete was mounted on the head as it required a direct line of sight to the satellites during the entire measurement sequence. This mounting point is considered a reasonable representation of the athlete as a point mass when the athlete is in the steady glide phase since there is little change in the athlete’s posture during this particular phase^3^. The raw position data from the dGNSS were filtered with a weighted cubic spline filter, where position error estimates from the geodetic dGNSS proceedings were used as weights^33,34^. All dGNSS data were translated and rotated from the global coordinate system WGS84 to a local coordinate system with the origin of the coordinate system set to the inrun edge with the horizontal direction denoted as the x-coordinates, and the vertical direction along the gravity vector denoted as the y-coordinates.

### Calculation of performance variables

From the spline-filtered dGNSS position data, velocity and acceleration were derived as time derivatives for the entire aerial phase^3^. The variables were filtered with a second-order Butterworth filter with cut-off frequencies for position data set to 2 Hz, the first derivative to 3 Hz and the second derivative to 2 Hz in accordance with Elfmark et al.^4^. These filtered 3-D vector data (position, velocity and acceleration) were then applied to calculate the following performance variables. The trajectory was applied to assess the athletes’ altitude at a given distance along the x-axis. Horizontal velocity, vertical velocity and aerodynamic variables were averaged for each athlete.

The aerodynamic drag and lift forces are defined as forces acting parallel and perpendicular to the direction of motion (DoM)^35^. The lift ($F_L$) and drag ($F_D$) force are defined as

1 $$\begin{aligned} F_{L,D} = ma_{L,D} = \frac{1}{2}\rho v^2C_{L,D}A, \end{aligned}$$

where *m* is the mass of the athlete, $a_{L,D}$ is the lift/drag acceleration, *v* is the velocity relative to the DoM, $\rho$ is the air density, $C_{L,D}$ the lift/drag coefficient and *A* the projected frontal area. The aerodynamic accelerations $a_{L,D}$ are calculated from the horizontal (*x*-direction) and vertical (*y*-direction) accelerations derived from the dGNSS and the angle between the direction of motion (velocity vector) and the global coordinate system ($\varphi$) as

2 $$\begin{aligned} a_L = -\ddot{x}\sin (\varphi )+(\ddot{y}+g)\cos (\varphi ) \end{aligned}$$

and

3 $$\begin{aligned} a_D = \ddot{x}\cos (\varphi )+(\ddot{y}+g)\sin (\varphi ). \end{aligned}$$

The aerodynamic accelerations (forces) have an exponential relationship with velocity [Eq. (1)]; thus, the aerodynamic properties are highly influenced by *v*. Hence, lift and drag, extended from variables, are defined as

4 $$\begin{aligned} \Omega C_{L,D}A = \frac{a_{L,D}}{v^2}, \end{aligned}$$

where $\Omega =\frac{\rho }{2m}$, is used to investigate the aerodynamic properties that are not influenced by the velocity, similar to Elfmark et al.^31^. The variables $C_LA$ (lift area) and $C_DA$ (drag area) are normally used in sports aerodynamic research^18,19,36^; however, this was not possible in this study as mass and air density were unknown. The lift-to-drag ratio (*LD*-ratio) was therefore defined as $C_L$/$C_D$ since the air density and body mass then become identical.

The steady glide phase was defined as the phase where the rate of change in *LD*-ratio varied within ${0.01}\hbox {s}^{-1}$, found by an algorithm described by Elfmark et al.^4^. Glide preparation is the phase from when the athlete is airborne to the start of the steady glide, and landing preparation is the phase from the end of the steady glide until the athlete reengages with the ground. The reader is referred to Elfmark et al.^4,31^ for more information about the ski jumping phase definitions. To compare the athletes’ single jumps in the statistical analysis, the data (position and all calculated variables) for each jump were aligned on a fixed increment along the x-axis as described in the supplementary material of Gilgien et al.^37^. The main analysis was based on the section of the steady glide phase where all jumps included in the analysis for all athletes were steady. Hence, the region from 20.5–87 m was analyzed. The horizontal length from the take-off, measured with the dGNSS, was used as the length measurement. This was more accurate than the video measurement system provided by FIS and comparable to the other variables measured with the same system. The average jump length, start of steady glide, end of steady glide, and take-off speed for the three athletes are presented in the first part of the “Results” section.

### Statistical analysis

A one-way repeated measures ANOVA with Tukey’s multiple comparisons test ($\alpha$ = 0.05) was applied to the average variables of jump length, start of steady glide, end of steady glide, percentage steady glide of jump length and take-off speed of the three athletes, to determine differences in the overall performance.

The trajectory, horizontal velocity, vertical velocity, and aerodynamic variables were averaged for each athlete and analyzed from 20.5–87 m, the region where all jumps were in the steady glide phase. The statistical analysis of these variables was performed using statistical parametric mapping (SPM) with an open-source software package SPM-1D (1-dimensional SPM, www.spm1d.org; ©T.C. Pataky, Accessed: 08.03.2021). Here, a t-test is executed for every time point of the average of each athlete, and random field theory^38^ is used to calculate a threshold test value (based on smoothness of data and the significance level) and an overall *p*-value for supra-threshold clusters, compared to computing a *p*-value for every point. The three athletes were compared two and two in the SPM software. In the reporting of results, WC1 was assigned the color blue, WC2 red, and COC1 black. The colors of the athletes were mixed when they were compared: purple for the comparison of WC1 and WC2, dark blue for WC1 and COC1, and dark red for WC2 and COC1. SPM was also used by Elfmark et al.^3^ and is a relatively new statistical technique in sports biomechanics. SPM allows a graphical representation of statistical analysis of continuous data, which can make the interpretation of such data easier^39^. The analysis was performed in Matlab R2019b and the significance level was set to $\alpha$ = 0.05. Statistical differences between data are represented by purple (WC1 vs. WC2), dark red (WC2 vs. COC1) and dark blue (WC1 vs. COC1) bars in the regions where statistically different thresholds were found.

## Results

To provide an overview of the jumps for each athlete, the average jump length, and the distance at which the steady glide phase started (Start glide) and ended (End glide) are shown in Table 1. Significant differences were found for length and take-off speed between WC2 and the two other athletes, as indicated in the table. For the other variables, no significant differences were found between athletes, and hence the performance level of the jumps was relatively similar, especially the start and end location of the steady glide phase, and the length of the steady glide phase.

**Table 1:** Table shows the average for the variables of jump length (Length), distance along the x-axis from the take-off edge where the steady glide phase started (Start Glide) and ended (End Glide), the fraction of the distance where athletes were in steady glide phase compared to the jump distance, in % (% glide) and the take-off speed (Take-off speed) for each athlete.

Athlete	N	Length (m)	Start glide (m)	End glide (m)	% glide (%)	Take-off speed (m s^–1^)
WC1	9	101.2 ± 3.7**	19.8 ± 1.0	91.5 ± 4.7	69.9 ± 2.8	25.9 ± 0.1**
WC2	9	107.9 ± 3.6	20.3 ± 1.6	95.5 ± 4.7	69.7 ± 2.1	26.2 ± 0.2
COC1	6	99.8 ± 2.7**	19.9 ± 1.5	89.8 ± 3.6	70.1 ± 2.0	25.9 ± 0.1**

**Indicates a significant difference between the marked athlete and WC2 (p < 0.001).

**Figure 1:**
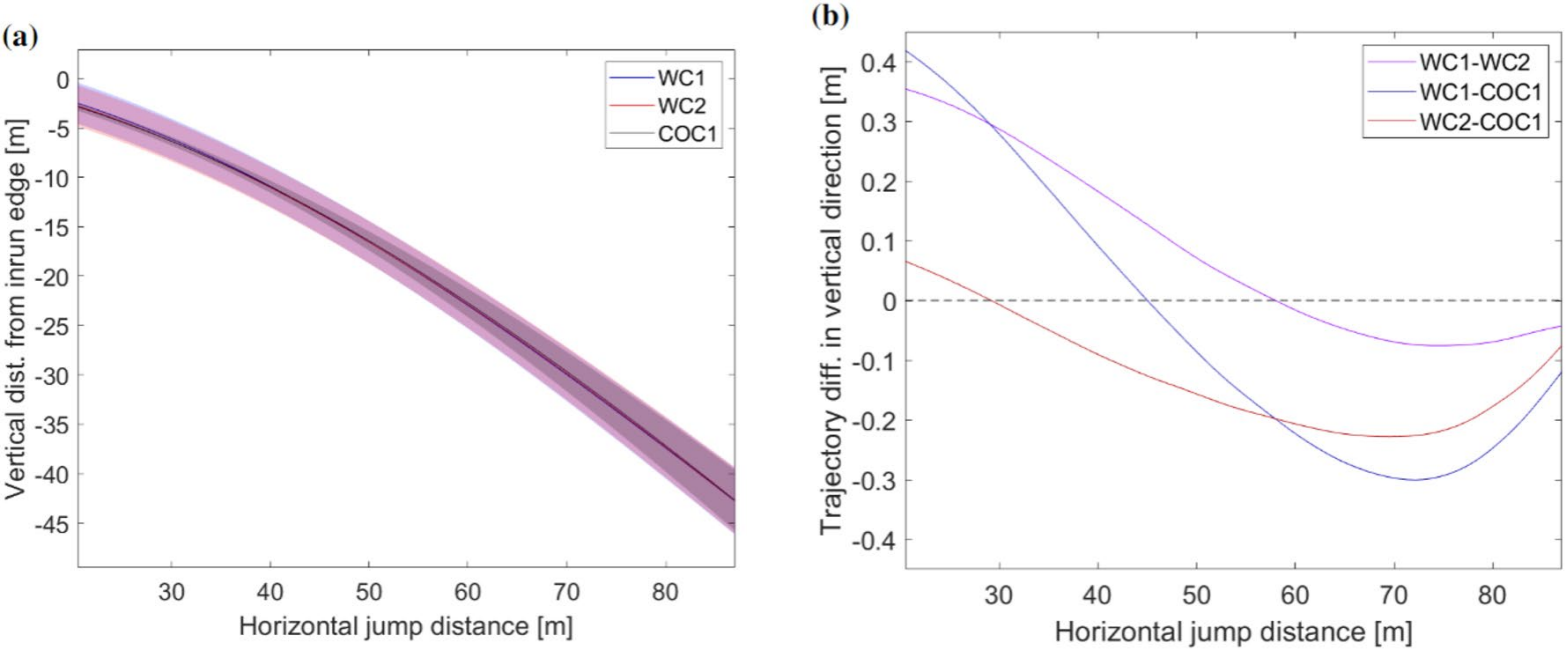
Average trajectories of the three athletes are shown in (**a**) and the trajectory differences in vertical direction between the athletes are shown in (**b**). The horizontal distance relative to the take-off edge is shown on the *x*-axis in both figures, vertical distance to the take-off edge is shown on the *y*-axis in (**a**) and trajectory differences in the vertical direction in (**b**). WC1, WC2 and COC1 are indicated by blue, red and black, respectively in (**a**) and the colors of the two that are compared in (**b**), indicated by purple, dark blue and dark red.

The following analysis is based on the continuous data from the fraction of the steady glide phase where all jumps were steady, which was the region from 20.5 to 87 m along the horizontal x-axis after the take-off edge. No significant differences were found between the athletes’ vertical positions. The average trajectories of the athletes can be seen in Fig. 1a, and differences in average trajectory between the athletes can be seen in Fig. 1b.

**Figure 2:**
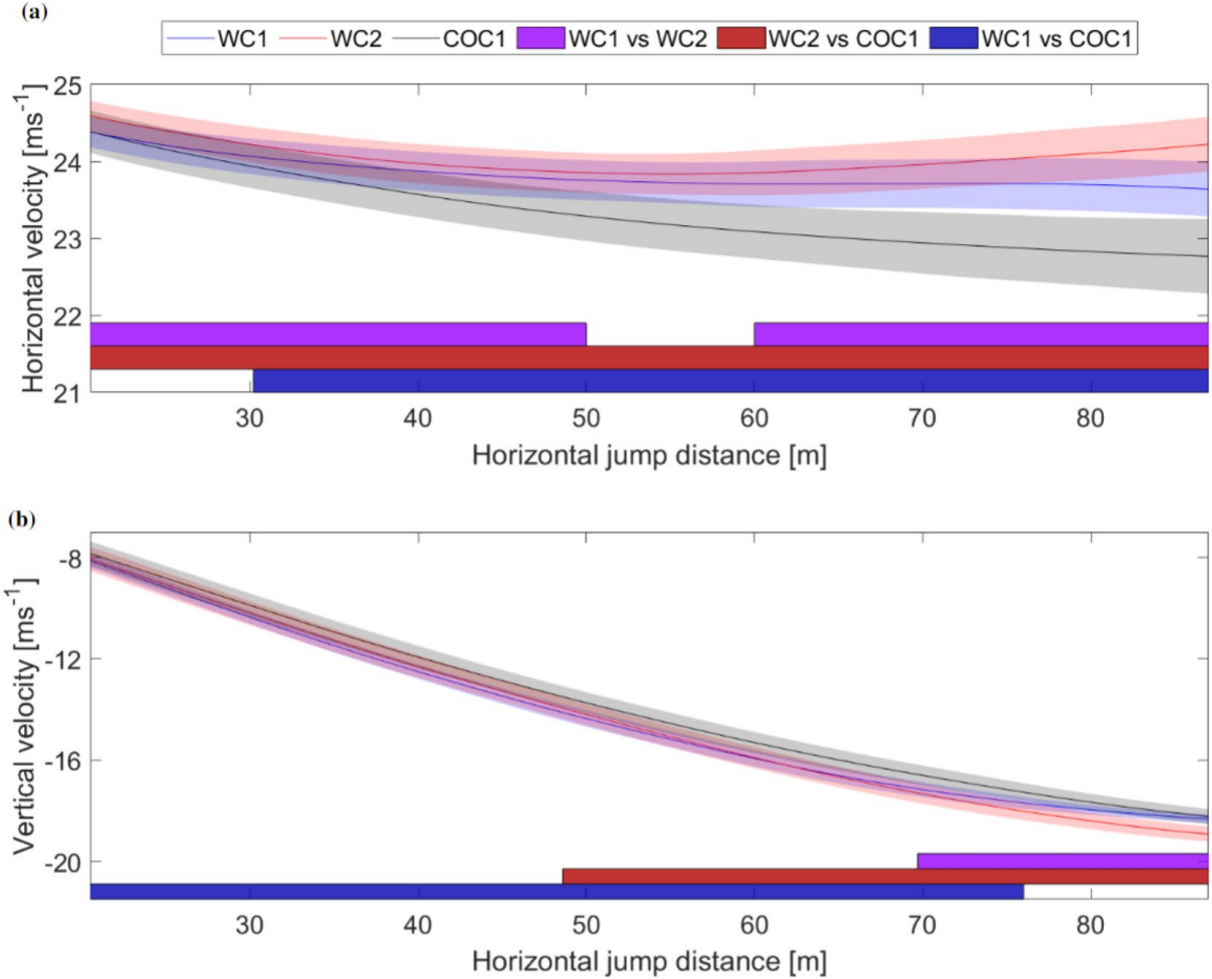
Continuous data for the average horizontal (**a**) and vertical (**b**) velocity for the three athletes. WC1, WC2 and COC1 are shown in blue, red and black respectively. Regions with significant differences from the SPM analysis are shown with colored bars (purple, dark red and dark blue) at the bottom of the plots with $\alpha$ = 0.05 and (p < 0.01). The horizontal distance relative to the take-off edge is shown on the *x*-axis and the velocity on the *y*-axis in both plots.

Although there were no significant differences between the trajectories of the athletes, there were some interesting trends. In general WC1 started the steady glide phase higher than WC2 but this difference evened out through the phase. WC1 also started off 0.4 m higher than COC1, but they crossed trajectories during the steady glide phase and COC1 was almost 0.3 m higher than WC1 after 70 m. Figure 2 shows the horizontal and vertical velocity components during the steady glide phase. Regions with significant differences between each athlete were found in both components.

The largest differences in velocity between the athletes were found in the horizontal direction. The horizontal velocity decreased for all athletes in the first part of the steady glide. The WC athletes managed to stall this decrease, while the COC1 athlete’s horizontal velocity decreased through the steady glide. WC2 had a significantly higher horizontal velocity than COC1 in the entire steady glide phase. WC2 also had a higher horizontal velocity than WC1 except in the section from 50 to 60 m. An interesting finding is that WC2 not only managed to stop the decrease of the horizontal velocity but also increased it through the latter part of the phase.

In the vertical velocity component, the individual differences may be more challenging to observe as the velocity component decreased from – 8 to – 20 m s^–1^. However, the SPM analysis also revealed differences between the three athletes in terms of the development of vertical velocity. COC1 had a lower vertical velocity than WC1 from the start of the steady glide to 76 m, and from 48.6 m to the end compared to WC2. The vertical velocities of the two WC athletes were generally more similar, with the only difference being that WC1 halted the decrease in the vertical velocity from 69.7 m, resulting in a lower vertical velocity from this point to the end of the steady glide, compared to WC2.

**Figure 3:**
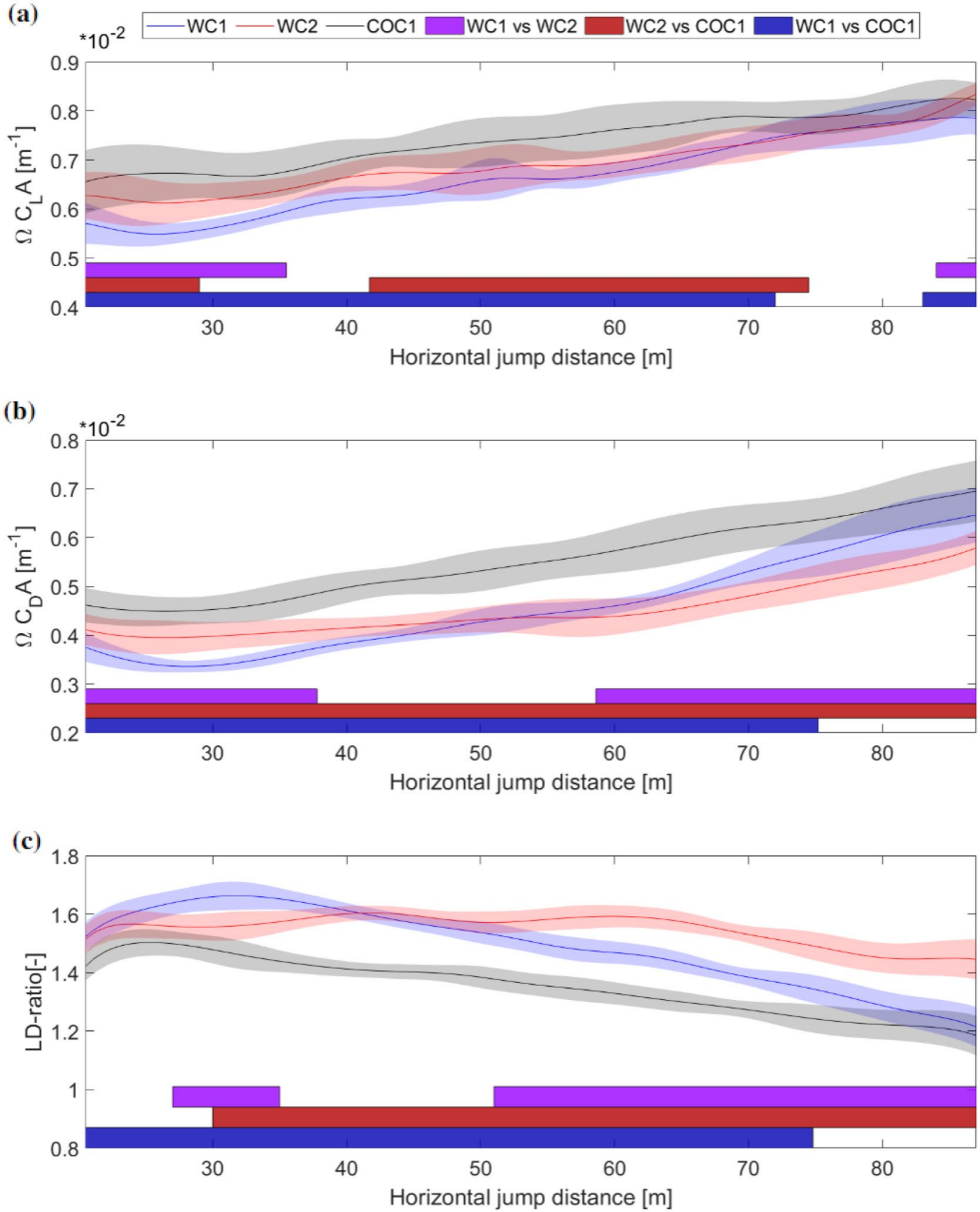
Continuous data of average (**a**) lift area ($\Omega C_LA$), (**b**) drag area ($\Omega C_DA$) and (**c**) *LD*-ratio for the three athletes. WC1, WC2 and COC1 are indicated by blue, red and black respectively. Regions with significant differences identified by the SPM analysis are shown with colored bars (purple, dark red and dark blue) at the bottom of the plots with $\alpha$ = 0.05 and (p < 0.01). The horizontal distance from the take-off edge is shown on the *x*-axis in all figures. Lift and drag area are shown on the *y*-axis in (**a**) and (**b**) and the dimensionless *LD*-ratio on the *y*-axis in (**c**).

Large regions with statistical significance between the athletes were also found in the aerodynamic variables (Fig. 3).

COC1 generally had higher aerodynamic forces and the lowest *LD*-ratio during the steady glide. WC1 had smaller aerodynamic forces in the first part of the steady glide phase and a region with a higher *LD*-ratio, compared to WC2. However, the largest difference between the WC athletes was observed in the last part of the steady glide phase (from 51 m), where WC2 had a higher *LD*-ratio. WC2 managed to maintain the high *LD*-ratio by stalling the drag force compared to WC1 in this region.

## Discussion

The purpose of this study was to investigate how different strategies in the steady glide phase can lead to similar performance, and how a superior strategy can lead to improved performance. The steady glide phases started and ended at the same distance from the take-off, and hence were similar in length and location. Even if the trajectories (in the x- and y- plane) of the three athletes were statistically similar, clear differences were found in the velocity components and aerodynamic variables between the athletes. This indicates that the three athletes in this study had their own distinct solutions, which led to similar performance levels for two athletes and superior performance for the third athlete (WC2).

Clear individual differences were observed between the three athletes for all of the aerodynamic variables in Fig. 3. COC1 had the highest lift and drag area and the lowest *LD*-ratio during the main part of the steady glide phase. One explanation for these differences in aerodynamic forces could be that COC1 might have had a larger angle of attack during the steady glide phase. This hypothesis is in line with previous findings on how the angle of attack affects the aerodynamic variables, where an increase in the angle of attack increases the aerodynamic forces while the *LD*-ratio decreases^18,40^. WC1 started off with the lowest aerodynamic forces and the highest *LD*-ratio, but had the steepest decrease in *LD*-ratio from ~ 30 m to the end of the steady glide phase, explained by the steepest increase in drag force. WC2 managed more or less to maintain the *LD*-ratio during the steady glide phase and had the highest *LD*-ratio from ~ 44 m, mainly by having a lower drag force than the two others. These findings are in line with Elfmark et al.^31^ who found that a high average *LD*-ratio had a substantial influence on performance on a large hill. This was achieved by maintaining lift and reducing drag. Although there were clear differences between all three athletes, the two WC athletes were in general more similar in that they generally had lower forces and higher *LD*-ratios in the steady glide phase.

The relationship between the aerodynamic forces and the velocity components was recently explained by Elfmark et al.^31^. For a ski jumper to achieve good performance during the steady glide phase, the athlete would aim to: I. Oppose the vertical acceleration as much as possible, and; II. Maximise horizontal velocity. I. can be achieved by having high aerodynamic forces and II. by having a high *LD*-ratio. It has previously been hypothesized that athletes normally favor one of these approaches since balancing or optimizing them is difficult, especially taking into account different hill sizes, which require different approaches^31^. This was also observed here on an individual level. COC1 had the highest aerodynamic forces during the steady glide phase, which lead him to halt the decrease of his vertical velocity compared to the WC athletes. However, this came at the cost of horizontal velocity as he lost horizontal velocity during the entire phase while the WC athletes maintained or increased it. The same argument can also explain the difference in velocity between the two WC athletes. WC1 had the lowest forces in the first part and also stalled the decrease of the horizontal velocity earlier than WC2. WC2 on the other hand had the advantage of a higher horizontal velocity from the inrun and had in general a higher *LD*-ratio during the steady glide phase, which not only halted the decrease of the horizontal velocity but increased it from ~ 60 m, again coming with the cost of a higher vertical velocity compared to WC1.

The fact that the aerodynamic forces are important in ski jumping is well known^2,18,22^, but the horizontal velocity data from WC2 show this in a remarkable manner. They show that a ski jumper can not only maintain but increase horizontal velocity during the steady glide phase by manipulation of the aerodynamic forces, which has never been addressed or shown in this manner before. WC2 was doing this consistently during his jumps. This can, to some extent, be explained by a study by Elfmark et al.^31^ showing that changing the horizontal velocity can be done by reducing the angle of attack, which in turn reduces the *LD*-ratio. Even though there were clear differences between all three athletes, the largest difference was seen between the WC athletes and the COC athlete, especially in the horizontal velocity. This can again be explained by the work of Elfmark et al.^31^. The COC athlete was more used to jumping on normal hills, or even smaller hill sizes. Here, the variables that are most important for good performance are to minimize vertical velocity, which is achieved by having a larger angle of attack and larger forces during the glide. WC athletes are used to jumping on large hills and ski flying hills, where maximizing horizontal velocity is most important, which can be established by a high *LD*-ratio. There are clear differences in jumping strategies between the two WC athletes, yet both of them have won individual WC competitions. Large differences in strategies were also found between WC1 and COC1, but there was no difference in their jump length. However, Elfmark et al.^31^ indicate that the technique of the COC1 athlete may be better for smaller hill sizes and not advantageous on large hills and in ski flying. Hence, ski jumpers must adapt their strategy to the hill size to enhance performance^41^.

WC2 jumped on average 7 m longer and had 0.3 m s^–1^ higher take-off speed than the two other athletes. However, no differences were found in the average start and end point of the steady glide, or in the percentage glide of the aerial phase. This is explained by the fact that WC2 started the steady glide phase 0.4 m later and had a slightly shorter percentage glide phase than the two other athletes. This indicates that even if there were some differences in the performance of WC2 compared to the two other athletes, their jumps were generally similar. The main part of the analysis was also done on the steady glide phase, which showed no differences. Despite the fact that no statistical differences were found between the trajectories, a vertical position difference of approximately 0.4 m at the start of the steady glide phase was found, which is considered an advantage in ski jumping. To gain this vertical advantage, there is a possibility that WC1 produced more vertical force in the take-off than the two other athletes. However, this was not confirmed as the dGNSS is not accurate enough to measure kinetics and kinematics during the take-off phase, which could have been achieved by video annotation or IMU technology^3,13,14^. However, WC1 lost this advantage during the first half of the steady glide phase compared to the two other athletes. The difference in *LD*-ratio may explain how WC2 evened out the trajectory difference compared to WC1. COC1 generally had a lower vertical start than WC1 and a slightly different ability to maintain vertical height compared to the two other athletes.

For the trajectories, no significant differences were found between the three athletes, while each athlete showed distinct trends on a scale of some decimeters. This emphasizes the importance of using a measurement system such as a dGNSS to accurately measure the differences between athletes in terms of position and its derivatives, velocity and force, during the aerial phase. It is only possible to detect these individual strategies for jumps with similar performance by examining the velocities and forces of the jumps. Hence, the introduction of dGNSS in ski jumping research has enabled us to investigate the physics of the aerial phase in ski jumping with high internal and external validity. It also shows that measuring all parts of the task performed is important in understanding performance. While previous research has emphasized the importance of the take-off in ski jumping^2,6^, this study has shown that performance in the steady glide phase can also have a substantial effect on jump distance.

As in all outdoor sports, weather conditions can influence performance. This is particularly true for ski jumping, as performance is affected by wind conditions^42,43^. To minimize this factor, all jumps were performed consecutively by the athletes on the same days, in the same sessions, and from the same gate. The athletes were only allowed to jump when their coach gave them a start signal, allowing the coach to hold an athlete if the wind conditions were not optimal. Since no wind measurements were taken during the data collection, it is therefore unknown how wind affected the results, which is an apparent limitation of the study. However, as the athletes were measured simultaneously during the training sessions, the wind conditions were likely similar. One evident limitation in the present study is the lack of air density and mass measurements, since this affects the absolute values of lift and drag area. Consequently, the absolute values in the lift and drag area (Fig. 3) of the athletes were not precise since the mass was eliminated and would be different for each athlete. However, the trend was correct and since the mass difference between athletes at both WC and COC levels is very little, this would not change the results much^44^. This also means that a direct comparison with other studies is not possible. A possible limitation is that the athletes needed to carry the dGNSS receiver, with the antenna mounted on their head during the entire session, to enable the data collection. However, the athletes reported no discomfort in using the system and could perform the jumps as usual. In future work, the steady glide phase should be assessed on larger and smaller hills, with more athletes and a larger range of performance levels. Especially important is the inclusion of females to study the sex-specific aspects of ski jumping. With further technological enhancement, these sensors may also, in time, be small enough to use in competition to increase the external validity further.

## Conclusion

This study investigated individual differences in the steady glide phase in terms of kinematic and kinetic characteristics. No differences between athletes were found for the trajectory. For the velocity components and the aerodynamic variables significant differences were found between athletes. The differences in these variables were clearly larger between athletes than the variability between jumps from the same athlete. The largest difference was found between the WC athletes and the COC athlete. The WC athletes increased their *LD*-ratio by reducing their aerodynamic forces, and as an outcome their horizontal velocity increased. In contrast, the COC athlete had larger aerodynamic forces during the glide to minimize the vertical velocity. These differences could result from the different hill sizes COC and WC compete in. WC athletes compete in large hill and ski flying, and COC athletes in normal and smaller hill sizes. Hence, this article shed some light on the importance of strategy in the steady glide phase but also revealed that the two WC athletes differed substantially in their strategy and outcome. Perhaps the most interesting finding was that one of the WC athletes managed to increase their horizontal velocity during the steady glide phase, by manipulating the aerodynamic forces; i.e., the *LD*-ratio.
